# Is the Act of Compelling Vaccination Unconstitutionial?

**Published:** 1892-11

**Authors:** Thomas M. Wyatt

**Affiliations:** New York, N. Y.


### IS THE ACT COMPELLING VACCINATION UNCONSTITUTIONAL?

JUDGE THOMAS M. WYATT, NEW YORK, N. Y.

The session laws of the State of New York provide, L. 1860, ch. 438, § 1, that

"The trustees of the several common-school districts in this State, and the proper local boards of common school government in the several cities of this State, are hereby directed and empowered, under the provisions hereinafter set forth, to exclude from the benefit of the common schools therein any child or any person who has not been vaccinated, and until such time when such child or person shall become vaccinated."

The Fourteenth Amendment to the Constitution of the United States, provides:

"SECTION 1. All persons born or naturalized in the United States, and subject to the jurisdiction thereof, are citizens of the

United States and of the States wherein they reside. No State shall make or enforce any law which shall abridge the privileges or immunities of citizens of the United States; nor shall any State deprive any person of life, liberty, or property, without due process of law, nor deny to any person within its jurisdiction the equal protection of the laws." And further,

Section 6 of the first article of the Constitution of the State of New York provides, that,

"No person shall be deprived of life, liberty, or property without due process of law."

Every citizen is called upon to pay and does pay, directly or indirectly, a school tax for the purpose of maintaining the public schools; in consideration of said tax so imposed and paid, he is to receive, and there is guaranteed to him, certain school benefits and privileges, and the free common schools of the country for the benefit and education of all citizens alike are the result. And to deprive a citizen of the benefit of the public schools for which he has paid, is to deprive him of the privileges and immunities guaranteed to him by the Constitution of the United States and of the State of New York without due process of law.

The laws of the State of New York compel the parent or guardian under certain pains and penalties to send every child over whom he has any control to the common schools, and at the same time the law deprives that child of the privilege and benefits of such school unless the parent or guardian has complied with certain regulations.

The Act of 1860, of the State of New York, is in the nature of a fine or penalty, by which certain persons, who are citizens under the Constitution of the United States and of the State of New York, are deprived of certain privileges, immunities, and benefits without due process of law.

That no citizen shall be affected in person or property by any proceeding in which he has not had an opportunity to be heard, lies at the foundation of all law of civilized and enlightened nations. There can be no due process of law without opportunity of defense. Notice to appear and protect his interest is the essential privilege of every citizen of the United States.

"Constitutional provisions declaring that no person shall be deprived of life, liberty, or property without due process of law is not limited to judicial proceedings which may interfere with those rights, whether judicial, administrative, or executive" (Stuart *vs.* Palmer, 74 N. Y. 183).

The statute of the State of New York first above quoted, creates the trustees of the several school boards of the several school districts and the local boards of common school government of the several cities of the State a quasi court to determine the eligibility of every child or person applying for the privileges of the common schools and may allow or deprive the child or person of that privilege or advantage. The rights above stated are determined without notice, hearing, or appeal. There is no requirement laid down in the statute requiring notice to be given to said child or person, or parent or guardian, or allowing a hearing or trial to be had affecting the rights of said citizen to be adjudged thereby. Thus the decision of the above constituted court is made and judgment given without due process of law.

In The People *vs.* The Board of Health, 58 Hun, 595, it was decided that had the statute under which the Board was authorized to require a railroad company to make openings in an embankment, dispensed with the necessity of a notice, the act would have been unconstitutional, the duties of the Board being quasi judicial in their nature.

In Chicago *vs.* Minnesota, 134 U. S. 418, Supreme Court of the United States declared an act of the legislature unconstitutional which authorizes the Board without notice to regulate the charges of the railroad company "as depriving the company of its property without due process of law and depriving it of the equal protection of the laws." (See also Clark *vs.* The Mayor, 13 Barb. 32 ; Babcock *vs.* The City of Buffalo, 1 Sheld. 317.)

Then it is plain to be seen that as the State, purporting to give power to the above-named school boards to determine the rights of certain persons, citizens, without notice, is unconstitutional and void.

No question can be raised questioning the fact that to deprive a person of a right or privilege is to deprive him of property, as a right is property, especially when that right is established, and is an advantage only obtainable by pecuniary consideration already paid, as in the case under consideration.

It cannot be claimed that this law is a police regulation. The police power of the State is not above and beyond the Constitution. The State legislation is at all times subject to the paramount authority of the Constitution, and cannot violate rights secured and guaranteed by the Constitution of the United States. (See 123 U. S. 663; Powell *vs.* Pennsylvania, 127 U. S. 678.) This is well established in Clark *vs.* Mayer, 13 Barb. 36.

"The police power has never yet been fully described nor its extent limited further, at least, than this: it is not above the Constitution, but is bounded by its provisions, and if any liberty or franchise is expressly protected by any constitutional provision, it cannot be destroyed by any valid exercise by the legislature or the executive of the police power" (People *vs.* Gilson, 109 N. Y. 400).

"The police power, however broad and extensive, is not above the Constitution. When it speaks its voice must be heeded. It furnishes the supreme law, the guide for the conduct of legislatures, judges, and private persons, and, so far as it imposes restraints, the police power must be exercised in subordination thereto. * * * The power is not without limitations, and in its exercise the legislature must respect the fundamental rights guaranteed by the Constitution. If this were otherwise, the power of the legislature would be practically without limitations" (Matter of Jacobs, 98 N. Y. 108–110; People *vs.* Marx, 99 N. Y. 377).

Thus it is seen that an act to enforce vaccination in the public schools is not an act of police regulation. Without doubt the legislatures of the various States may enact certain regulations to protect the public health, so long as the enactments are kept within the limits of the Constitution of the United States and of this State; beyond that it cannot go. The State legislature could as well enact that the children of certain parents might attend the public schools, while it prohibits the children of other parents that privilege. This discrimination would be obnoxious to the sense of every American citizen and in violation of every constitutional right and of justice.

And, furthermore, the attempted legislation on the part of the State is not an attack upon an evil that exists, but upon a possibility of an evil. It is not aimed at the sick, but at the well. It subjects the well to pecuniary damage, but does not attempt to cure the sick. Its shafts are leveled not at a reality, but at a possibility—a possibility that may never happen.

The legislature of the State might as well compel every child or person to be treated with injections of the lymph of Dr. Koch, to protect them from possible consumption, or with the bi-chloride of gold of Dr. Keeley, to prevent the possibility of drunkenness. If legislation is proper in one case, certainly it is in the other. The remedy claimed in either case is quite as certain as in the other, and any attempted legislation in either instance, when brought before the proper tribunal, would be declared unconstitutional and void.

---

A motion was made by Dr. Hastings that the sessions be held from 10 A. M. to 1.30 P. M., 4 to 6 P. M., and 8 to 10 P.M., or later.

Adjourned to 4 P. M.

